# From Focused Ultrasound Tumor Ablation to Brain Blood Barrier Opening for High Grade Glioma: A Systematic Review

**DOI:** 10.3390/cancers13225614

**Published:** 2021-11-10

**Authors:** Luca Paun, Alessandro Moiraghi, Gianpaolo Jannelli, Aria Nouri, Francesco DiMeco, Johan Pallud, Torstein R. Meling, Shahan Momjian, Karl Schaller, Francesco Prada, Denis Migliorini

**Affiliations:** 1 Division of Neurosurgery, Department of Clinical Neurosciences, Geneva University Hospitals, 1205 Geneva, Switzerland; gianpaolo.jannelli@hcuge.ch (G.J.); aria.nouri@hcuge.ch (A.N.); torstein.meling@hcuge.ch (T.R.M.); shahan.momjian@hcuge.ch (S.M.); karl.schaller@hcuge.ch (K.S.); 2 Department of Neurosurgery, GHU Site Sainte-Anne, F-75014 Paris, France; alessandro.moiraghi@sfits.ch (A.M.); j.pallud@ghu-paris.fr (J.P.); 3 Université de Paris, 102-108 Rue de la Santé, F-75014 Paris, France; 4 Institut de Psychiatrie et Neurosciences de Paris (IPNP), UMR S1266, INSERM, IMA-BRAIN, F-75014 Paris, France; 5 Department of Neurosurgery, Fondazione IRCCS Istituto Neurologico “C. Besta”, 20133 Milan, Italy; fdimeco1@jhmi.edu (F.D.); francesco.prada@istituto-besta.it (F.P.); 6 Department of Pathophysiology and Transplantation, University of Milan, 20122 Milan, Italy; 7 Department of Neurological Surgery, Johns Hopkins Medical School, Baltimore, MD 21205, USA; 8 Faculty of Medicine, University of Geneva, 1206 Geneva, Switzerland; 9 Focused Ultrasound Foundation, Charlottesville, VA 22903, USA; 10 Department of Neurological Surgery, University of Virginia, Charlottesville, VA 22903, USA; 11 Department of Oncology, Geneva University Hospitals, 1205 Geneva, Switzerland; 12 Center for Translational Research in Onco-Hematology, University of Geneva, 1211 Geneva, Switzerland

**Keywords:** focused ultrasound, high grade glioma, glioblastoma, brain-blood barrier opening, tumor ablation, ultrasound, targeted drug delivery

## Abstract

**Simple Summary:**

High-grade glioma (HGG) is a burdening oncological pathology for which maximum safe resection followed by combined chemoradiation therapy is still the gold standard. Despite these treatments the overall survival is less than 2 years. Alternative strategies are currently being investigated, including Focused Ultrasound (FUS). Given its non-invasiveness and promising pre-clinical results for tumor ablation, brain-blood barrier (BBB) opening and drug delivery, FUS is poised to achieve a therapeutic role in HGG treatment. This systematic review aims to identify the different modalities of how FUS can be used, how it impacts on survival in the clinic, based on the existing evidence. FUS-mediated tumor ablation still needs further investigation due to its controversial effects and complications. FUS-mediated BBB opening is showing positive results with low complication rate, as such potentially a gamechanger in future oncological treatments. Ongoing trials will clarify FUS impact on HGG patients.

**Abstract:**

Background: Focused Ultrasound (FUS) is gaining a therapeutic role in neuro-oncology considering its novelty and non-invasiveness. Multiple pre-clinical studies show the efficacy of FUS mediated ablation and Blood-Brain Barrier (BBB) opening in high-grade glioma (HGG), but there is still poor evidence in humans, mainly aimed towards assessing FUS safety. Methods: With this systematic review our aim is, firstly, to summarize how FUS is proposed for human HGG treatment. Secondly, we focus on future perspectives and new therapeutic options. Using PRISMA 2020 guidelines, we reviewed case series and trials with description of patient characteristics, pre- and post-operative treatments and FUS outcomes. We considered nine case series (five about tumor ablation and four about BBB opening) with FUS-treated HGG patients between 1991 and 2021. Results: Sixty-eight patients were considered in total, mostly males (67.6%), with a mean age of 50.5 ± 15.3 years old. Major complication rates were found in the tumor ablation group (26.1%). FUS has been rarely applied for direct tumoral ablation in human HGG patients with controversial results, but at the best of current studies, FUS-mediated BBB opening is showing good results with very low complication rates, paving the way for a new reliable technique to improve local chemotherapy delivery and antitumoral immune response. Conclusions: FUS can become a complementary technique to surgical resection and standard radiochemotherapy in recurrent HGG. Ongoing trials could provide in the near future more data on FUS-mediated BBB opening impact on progression-free survival, overall survival and potential drug-delivery capacities.

## 1. Introduction

Ultrasonic waves are sound waves with a frequency > 20,000 Hz used for the first time during World War I for submarine detection [1,2]. During the 20th century progressively, ultrasound (US) started to be considered also as a medical tool able to diagnose and also to treat diseases such as autoimmune disorders [3,4] and started to be considered as a new tool to treat neurological pathologies: Lynn in 1942 and the Fry brothers in 1962 were the firsts to use this technology for tumor ablation [5,6,7]. At that time, the main problem impeding transcranial sonications for neuro-oncological purposes was the acoustic barrier represented by the skull, which lead to a loss of target accuracy and lack of temperature control; for these reasons these procedures were performed after bone flap removal [2,8,9].

During the past two decades, US has been widely used for intraoperative guidance and resection control in glioma surgery [10,11,12,13,14,15], in part due to its capacity to be complementary to other intraoperative techniques like 5-Aminolevulinic acid (5-ALA) fluorescence [16], intraoperative magnetic resonance imaging (MRI) [17], awake mapping [18,19,20] and asleep neuromonitoring [21,22,23].

Recently, the combination of stereotactic techniques, MRI and acoustic aberration phase correction permitted an increase in US accuracy [24,25]. High-intensity MRI-guided FUS has been used to induce a precise thermal ablation of the target tissue using a 650-Hz-frequency ultrasound energy, which can increase tissue temperature to 65 °C [26,27]. This technique led FUS to become an excellent alternative in functional neurosurgery (mostly for Parkinson’ disease and essential tremor), permitting non-invasive and rapid procedures [24,25]. New applications are being studied for neuromodulation and epilepsy with encouraging results [28]. The parallel introduction of transducers, water baths, degassed water and finally microbubbles considerably augmented the acoustic coupling, allowed a reduction of FUS-related side-effects such as thermal injury of the scalp and optic aberration of FUS waves [29,30]. Although promising, in neuro-oncology FUS direct ablation showed some limitations especially concerning a small treatment envelope and the time required to ablate largest tumors [31,32,33].

Different studies on animals and human models have been looking at FUS’ capabilities in high grade gliomas (HGG) ablation and in brain-blood barrier (BBB) opening in order to maximize the penetration of neo-adjuvant and adjuvant treatments to the central nervous system [34,35,36]. As we know BBB restricts and controls water-soluble substances movement between the blood and the parenchyma. This function can be altered in HGG, where endothelial cells lose their intercellular tight junctions and ability to prevent water-soluble molecules entry in the tumor, whilst permits a tumoral protection in chemotherapy delivery [37,38]. In fact, most chemotherapy drugs have large molecular weights, and considering BBB semi-permeability and selectiveness, the latter becomes a limiting obstacle in brain tumor treatment [39]. Multiple preclinical and clinical studies in animal glioma models have evaluated the safety and efficacy of BBB disruption with FUS [40,41,42]. Different large animal studies showed a possible drug delivery with FUS aid, especially concerning trastuzumab, doxorubicin, temozolomide (TMZ), methotrexate and carboplatin [39].

With this review, our aim is to summarize how FUS has been proposed for human HGG treatment as a useful technique to perform direct tumor ablation and BBB opening for chemotherapy. Secondarily, we point out which future perspectives and new therapeutic option are being achieved and studied with ongoing human trials.

## 2. Materials and Methods

### Search Strategy, Inclusion Criteria, and Study Selection

**Figure 1:**
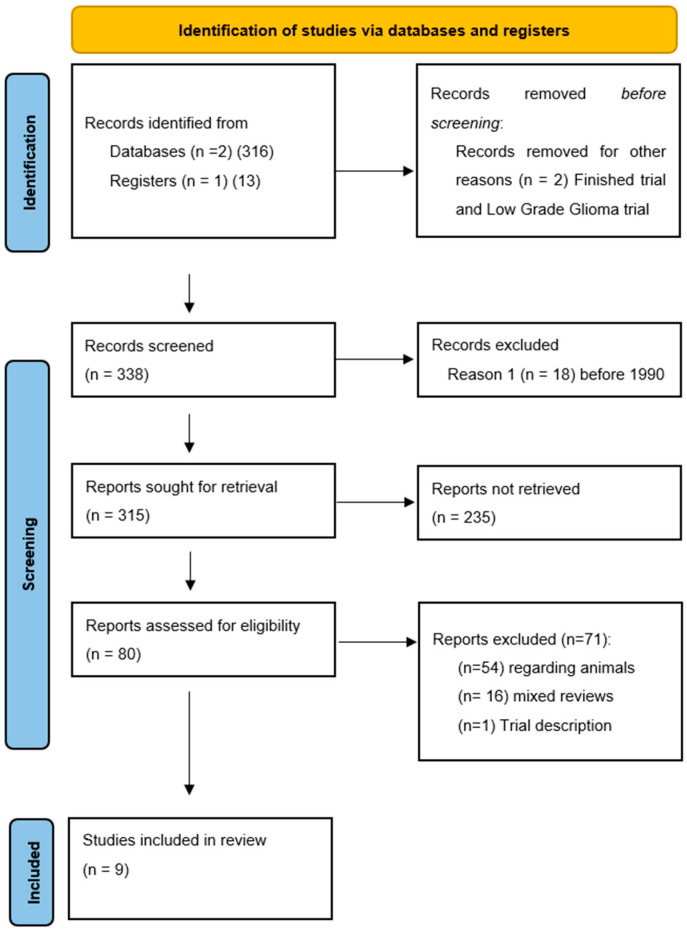
PRISMA-P 2020 flow-chart and search strategy. From: Page MJ, McKenzie JE, Bossuyt PM, Boutron I, Hoffmann TC, Mulrow CD, et al. The PRISMA 2020 statement: an updated guideline for reporting systematic reviews. *BMJ* **2021**, *372*, n71. doi:10.1136/bmj.n71. For more information, visit: http://www.prisma-statement.org/.

The study protocol followed the Preferred Reporting Items for Systematic reviews and Meta-Analyses (PRISMA-DTA) 2020 guidelines [43]. No PROSPERO registration was needed. We conducted a restricted search using the keywords “*Focused Ultrasound*” AND “*Glioma*” in May 2021 of the following databases: PubMed/Medline, Google Scholar and clinicaltrials.gov. This resulted in a total of 338 references (Figure 1).

The first two authors (LP and AM) independently screened all titles and abstracts, and full-text copies of all relevant articles were obtained with exclusion of no pertinent studies. In case of a discrepancy, the senior author (DM) arbitrated until a consensus among the authors was reached. The following inclusion criteria were used: (1) All studies reporting single or multiple cases of HGG treated with focused ultrasound; (2) studies published since 1990, as the standards of neurosurgery has significantly improved since then and results before this era are not comparable. 

In total, 338 abstracts were screened, and 80 papers were retained for eligibility. During the review process, we searched for all reported cases where FUS was used on human patients harboring intracranial HGG. Research not involving HGG patients treated with focused ultrasound (5 articles) and before 1990 (18 articles) did not meet the inclusion criteria. Seventy-one articles were excluded, considering that 54 reported non-human research, 16 were mixed reviews on FUS in neurosurgery and one was a trial description. The references of the selected studies were checked to find all possible related articles (Figure 1). A large research was made on purpose at the beginning of the review process to prevent possible lack of eligible studies, which on a small pool of reported patients would have generated a major selection bias. No statistical analysis was performed.

## 3. Results

### 3.1. Patient Characteristics

We considered nine case series reporting patients treated for cerebral gliomas with FUS between 1991 and 2021. Sixty-eight patients were included (46 males and 22 females), of whom 63 benefitted from a previous treatment (surgery in 11 cases; radiotherapy in 52 cases) (Table 1). The mean age was of 50.5 ± 15.3 years (range, 17–80). Sixty-one patients harbored a glioblastoma, whilst seven had an anaplastic astrocytoma: all HGG were supratentorial. In our review, five papers included tumor ablation techniques whilst four analyzed BBB opening. 

### 3.2. FUS Devices for Direct Tumor Ablation

Guthkelch et al. [44,45,46] in 1991 used a radio-frequency signal that is amplified and converted to US by a curved piezoelectric transducer from a modified obstetrical ultrasound. Intracerebral temperatures were measured with flexible thermocouple probes introduced under CT-scan guidance through the craniotomized area.

In 2006, Park et al. [47] used transcranially the JC HIFU system (Chongqing HAIFU Medical Technology Co., LTD., Chongqing, China) on a 17 year-old female patient affected by an anaplastic astrocytoma. The patient had previously benefitted from a debulking through a craniotomy. The system is composed of a treatment table coupled to a high-frequency ultrasound and controlled by a central console. The US transducer can create a beam propagating through tissues with a temperature above 56 °C, provoking immediate thermal toxicity. 

A similar FUS machine, used in further studies, is the ExAblate^®^ (InSightec, Haifa, Israel). The latter consists in a 30 cm diameter hemispherical 512 up to 1024-element phased-array transducer coupled with a 512-channel driving system, a treatment planning workstation with MRI thermometry and dosimetry and a water cooling, circulation and degassing system, integrated with a clinical MRI Unit [33]. The US frequency is 620–720 KHz.

### 3.3. FUS Devices for BBB Opening

A French research consortium developed and used an implanted US device named SonoCloud^®^ (Carthera^®^, Paris, France), consisting of a 10 mm diameter US transducer that had a resonance frequency of 1.05 MHz and was encased in an 11.5 mm diameter biocompatible housing [48]. The combination with ultrasound-specific microbubbles (Sonovue^®^, Bracco Imaging, Milan, Italy) permits a lower FUS treatment intensity. In Toronto Mainprize et al. used ExAblate^®^ system to study chemotherapy BBB passage and concentration after its opening [49]. Finally Chen et al. [50] introduced a frameless neuronavigation US device called NaviFUS^®^ (NaviFUS Inc., Taipei, Taiwan), that integrated with microbubbles is able to steer in real-time the transcranial ultrasound energy precisely and repeatedly at targeted central nervous system areas.

### 3.4. FUS for Tumor Ablation

In our review, we identified 23 patients who benefitted from a FUS tumor ablation procedure (Table 2). Five patients (8%, all in Guthkelch’s series [44]) did not had any other treatment purposed apart from FUS. This population was younger compared to BBB opening cohort. Eleven patients benefited from a pre-FUS tumor debulking and 18 from radiotherapy. Karnofsky performance score (KPS) prior to FUS was assessed only by Guthkelch et al. [44] (Table 3).

The authors used different power and length of ultrasound sonication with a mean value of 30.3 ± 6.8 sonications per patient. In 16 patients the number of sonications was not reported [44,47]. Depending on different authors, target position, beam temperature and low-power verification sessions before sonications were applied, with a lower temperature that was augmented once the target was confirmed by MRI or Computed Tomography (CT)-scan co-registration or overlap. Temperature was adapted considering patient compliance, discomfort and/or neurological symptoms. The mean follow-up for 19 patients was of 19.7 ± 14.6 months (four patients did not have a specified time range). In tumor ablation cohort, six patients (26.1%) presented a major FUS-related complication (four deep hematomas, one subcortical hematoma and one unwanted lesion in an eloquent area) (Table 3). No neurological outcome at long term follow-up was reported. No data was found concerning tumor volume stability.

### 3.5. FUS-Mediated BBB Opening

Forty-five patients were included in this subpopulation, presenting a mean age of 56.6 ± 5.3 years. In all cases, BBB opening was performed to enhance post-surgical chemotherapy. No data was found concerning pre-FUS tumor debulking procedure (Table 4 and Table 5), whereas 17 patients had pre-FUS radiotherapy. Four patients (8.8%, all in Mainprize’s series [49]) were enrolled in a safety and feasibility study so they had FUS as first treatment before any surgery or chemo-radiotherapy approach. In three studies, the KPS was considered before FUS delivery with a limit respectively at 70–100 (Mainprize et al. [49]), 90 (Idbaih et al. [51]), and 90 (Chen et al. [50]). Ultrasound sonications were augmented in number and intensity considering patient tolerance et lack of adverse events with a mean number of 53 ± 17. The BBB opening in three studies (Carpentier et al. [48], Idbaih et al. [51], and Chen et al. [52]) was mediated by intravenous (IV) bolus injection of SonoVue^®^ microbubbles (Bracco Imaging, Milan, Italy) or by Definity microbubbles (Lantheus Medical Imaging, MA, USA). All the studies pointed to determine if BBB opening was safe, feasible, measurable, repeatable, and reversible without giving any major adverse event. French and Canadian collectives concurrently also administered chemotherapy (Carboplatin in Carpentier et al. [48] and Idbaih et al. [51], whilst Mainprize et al. [49] delivered Doxorubicin or TMZ). Chemotherapy delivery, penetrance and distribution still need further studies. Local inflammation and immunological induced response post-FUS were not explored. In those series no complications were found, and the temporary mean follow-up was of 13.7 ± 16.1 months. Progression-free survival (PFS) and overall survival (OS) were assessed only by Idbaih et al. [51] that demonstrated an increase of those parameters in FUS-BBB disruption (OS 12.94 vs. 8.64 months and PFS 4.11 vs. 2.73 months) compared to poor overture. 

### 3.6. Ongoing Clinical Trials

Thirteen clinical trials are studying the impact of FUS on HGG treatment (11 about BBB opening and two about tumor ablation) (Table 6). Most of these studies are mainly focused on FUS-mediated BBB opening to increase chemotherapy delivery and distribution. The new ongoing clinical trials are performed with SonoCloud^®^, Exablate^®^ and NaviFUS^®^. Zacharoulis et al. at Columbia University are evaluating FUS sonication with microbubbles aid to disrupt selectively and temporarily the BBB to augment oral Panobinostat concentration in children affected by diffuse midline gliomas (NCT04804709).

Sanai et al. at the Barrow Neurological Institute are using ascending doses of MR-guided FUS in combination with 5-ALA to assess safety and efficacity in recurrent HGG (NCT04559685). The measurement is made with Cleaved Caspase-3, MIB-1 level, GammaH2Ax in surgical specimen tissue. Another prospective study to be conducted at Besta Institute in Milan will evaluate the safety and feasibility of sonodynamic therapy with 5-ALA in patients with newly diagnosed GBM [53,54,55].

Sunnybrook Health Sciences Centre in Toronto is leading three different trials on tumor ablation, BBB opening and Carboplatin delivery through BBB (NCT01473485; NCT03616860; NCT04440358). A multicenter prospective trial between Baltimore, Boston, Charlottesville and Morgantown is investigating the safety and feasibility of periphery tumor cavity BBB disruption with adjuvant planned temozolomide infusion (NCT03551249). In Taiwan, a new prospective pilot study is investigating the efficacy and safety of FUS in BBB opening with complementary administration of bevacizumab in patients with recurrent glioblastoma (NCT04446416). Another multicenter collective, composed by teams in Palo Alto, Baltimore, Boston and Cleveland, is studying BBB disruption combined with IV carboplatin for recurrent GBM treatment (NCT04417088).

In Korea, Chang et al. are studying BBB disruption along the periphery of tumor resection cavity with a subsequent planned adjuvant TMZ chemotherapy (NCT03712293). Three new trials conducted with Sonocloud^®^ aid are studying drug delivery through BBB opening. A phase 1 and 2 trial driven by Carpentier et al. is evaluating Sonocloud^®^ efficacity on the dose limiting toxicity (DLT) of escalating numbers of ultrasound beams at constant acoustic pressure and its safety and efficacy of BBB opening (NCT03744026). 

At Northwestern University, Stupp et al. are evaluating with a phase 1 and 2 trial an intraoperative implantation of Sonocloud^®^ in order to open safely and repeatedly the BBB for Albumin-bound Paclitaxel chemotherapy (NCT04528680). The aim is to establish a safe and effective dose of Albumin-bound Paclitaxel through BBB opening. Finally, the multicentric SonoFIRST trial is studying if Sonocloud^®^ could improve the PFS of newly diagnosed GBM patients, treated by concurrent chemo-radiotherapy and adjuvant temozolomide compared with patients with standard of care alone (NCT04614493). 

## 4. Discussion

In our systematic review, we have selected all the studies reporting the results of FUS application in treating human HGG. As a matter of fact, our study demonstrates how very few studies exist in the literature (n = 9), and they are all preliminary experiences/trials conducted on small numbers of patients (n = 68).

### 4.1. FUS Direct Tumor Ablation

Five different studies investigated the effects of FUS in direct tumor ablation for treating HGG patients (n = 23). In this subpopulation, complication rates were high, with more than one patient out of four developing a hematoma in the targeted FUS area. However, this could be due to the use of early devices in a series of patients that did not benefit from previous knowledge regarding selection criteria and risk factors for this specific treatment. Only 6.4% of the patients had to stop FUS delivery due to complications, which represents an overall good tolerance during treatment delivery. The lack of data on patients’ neurological outcome at follow-up could be explained by the fact that most of the studies were case series reports or preliminary reports of ongoing trials. Future studies should provide details on patient selection and outcome and could possibly allow us to better select patients who can benefit most and with the lower risk of complications.

Guthkelch et al. [44] pointed out that post-craniectomy FUS is effective in causing necrosis within the adequately-heated tumor volume with a mean temperature higher than 42 °C. On the other hand, one limitation was the non-uniform power distribution that does not permit a non-uniform tumoral sonication. Ram et al. [32] showed how MR-guided FUS gave immediate changes in contrast-enhanced T1-, T2- and diffusion-weighted MRI scans, in addition to signs of thermocoagulation of the tumor confirmed by histological examination. Only three patients with GBM underwent MR-guided FUS thermal ablation. First, they had a craniectomy 7–10 days before sonication to get a bone window for better ultrasound transmission [32]. One of these patients had a lesion caused by thermal ablation of the normal surrounding brain parenchyma outside the target in the pathway of transmission of the ultrasound waves, leading to neurological deficits [32]. Park et al. [47] used FUS in one patient for whom any other therapeutic option was judged inadequate, showing a tumor volume and peripherical oedema diminution in the 6 months follow-up.

In 2010, McDannold et al. [33] managed, for the first time, to focus the ultrasound beams transcranially into the brain and visualizing heating with MR temperature imaging ExAblate 3000 system in three GBM patients. They were unable to reach a complete tumor ablation due to low power of FUS device (650–800 W), and the trial was stopped when a fourth patient suffered a cavitation induced fatal intracranial hemorrhage [33]. In 2014, Coluccia et al. [31] reported a case where a 63-year-old patient was treated for a centrally located recurrent GBM: intraoperative MR thermometry identified 17 of the 25 sonications as capable of thermocoagulation, with temperature between 55 °C and 65 °C. Immediate post-procedural diffusion-weighted MRI identified multiple bright lesions representing the thermally coagulated tissue in the targeted tumor volume, and during the follow-up the patient showed a neurological improvement of his pre-procedural deficits [31]. In tumor ablation papers, complete follow-up data are missing and complication rates seem high, a possible explanation could be that those were pioneering studies with small patient samples. For that reason, ongoing trials should validate the real potential of FUS ablation power.

The authors used different power and length of ultrasound sonication with a mean value of 30.3 ± 6.8 sonications per patient. In 16 patients the number of sonications was not reported [44,47]. Depending on different authors, target position, beam temperature and low-power verification sessions before sonications were applied, with a lower temperature that was augmented once the target was confirmed by MRI or CT-scan co-registration or overlap. Temperature was adapted considering patient compliance, discomfort and/or neurological symptoms. The mean follow-up for 19 patients was of 19.7 ± 14.6 months (four patients did not have a specified time range). In tumor ablation cohort, six patients (26.1%) presented a major FUS-related complication (four deep hematomas, one subcortical hematoma and one unwanted lesion in an eloquent area) (Table 3). No neurological outcome at long term follow-up was reported. No data was found concerning tumor volume stability.

### 4.2. FUS-Mediated BBB Opening

Despite abundant preclinical evidence indicating the efficacy of BBB disruption using low-intensity FUS for enhanced delivery of various chemotherapeutic agents and viral vectors to the CNS, evidence regarding safety and efficacy of this treatment method in humans with brain tumors remains limited. In our review, among the patients (n = 45) who received FUS-mediated BBB opening for treatment of cerebral HGG, no complications were found, and patients’ mean follow-up was 13.7 ± 16.1 months. Mainprize et al. [49] firstly demonstrated a possible safe and effective BBB transient opening with systemically administered chemotherapy with FUS and microbubbles before surgical debulking in a phase I single arm open label study. In fact, in this study five patients benefitted of TMZ (n = 4) or Doxorubicin (n = 1) administration with FUS, microbubbles injection (Definity^®^; Lantheus Medical Imaging, North Billerica, MA, USA) and MRI control. The authors demonstrated a radiographic evidence of an immediate 15–50% increased contrast enhancement on T1-weighted MRI at BBB level, without any significant ultrasound related clinical or radiological adverse event, validating the BBB opening. One hour prior to the procedure, the patient received the subtherapeutic dose of chemotherapy and in the following day, the patient had a tumor resection. The procedure was well-tolerated, with a successful opening of the BBB based on increased gadolinium enhancement at T1-weighted MRI [49]. Tissue liquid-chromatography mass spectrometry analysis demonstrated greater concentration of liposomal doxorubicin and oral TMZ in brain regions where BBB disruption occurred compared to areas without BBB disruption [49]. Ultrasound contrast agents, such as microbubbles, amplify focal heating during sonication and are used to reduce the time-averaged power needed during transcranial FUS ablation; for this purpose real-time passive acoustic mapping is really helpful to avoid unwanted cavitation [56,57,58,59].

Quantitative analysis of microbubbles is also helpful to understand their circulation and plan microbubble-mediated treatments [60]. The French Carthera group developed an implantable, low-intensity pulsed ultrasound device system that permits BBB disruption using pulsed ultrasound in combination with systemically injected microbubbles [48]. In 2016, Carpentier et al. [48] showed the preliminary results on 15 patients that benefitted from repeated sonication that permitted safe delivery of systemic chemotherapy with carboplatin. The BBB was disrupted at acoustic pressure levels up to 1.1 MPa without detectable adverse effects [48]. Subsequently, Ibdaih et al. [51] showed that patients who had a good post-FUS BBB disruption had an augmentation of PFS and OS. At least one sonication was achieved in 19 patients. BBB disruption was evaluated with contrast-enhanced T1-weighted brain MRI and was visible after 52 out of 65 ultrasound sessions [51]. The treatment was safe without serious adverse events or carboplatin related neurotoxicity. Finally, Chen et al. [50,52] reported promising results with NaviFUS^®^. In animal models, preliminary evidence of FUS-induced immune modulation is being provided as an additional therapeutic benefit by converting the immunosuppressive tumor microenvironment into an immunostimulatory tumor microenvironment [50,52]. On human patients, a safe, dose-dependent and auto-reversible BBB opening is achieved without major adverse events and with a simple and easy-to-use machine [50,52]. NaviFUS^®^ seems to be extremely reliable for neurosurgeons as it does not need for a head-holder and a rapid trajectory planning can be performed with the aid of standard neuronavigation [50,52].

### 4.3. Ongoing Trials and Future Perspectives

To the best of our knowledge, most of the trials are involving adult patients and especially recurrent cases of GBM. BBB opening seems the most investigated subject. The promising results of French and Taiwanese collectives could pave the way for new treatment definition in HGG. Furthermore, US-guided therapy, as for general radiology, might be an option for brain therapy in the future: US transparent cranial prosthesis could be implanted after tumor resection in order to allow both diagnostic US-direct imaging alone, or as a guidance for FUS mediated therapies [55]. FUS implementation could, in fact, change in a cost-effective way new protocolled second-line treatment.

Ongoing trials should give concrete data on clinical status at long-term follow-up, validating the true ratio of adverse events. Even if excellent results were shown on animal models in post-FUS immunological acquired and innate awakening, major investigations should analyze this matter. A thoughtful consideration should be made regarding the therapeutic agents that are most likely to benefit from BBB opening to potentially make an oncological difference in future clinical studies: in fact, administration should be fast, easy, reproducible and effective.

The necessary repeated administration of the molecule should in particular be taken into account, permitting an effective and precise drug-delivery through the BBB.

Finally, two emerging preclinical applications of FUS could represent a future application in human brain tumor treatment. The first one takes advantage of BBB opening (using FUS in combination with microbubbles) to enhance the release of biomarkers from the brain tumor to the blood circulation, thereby allowing FUS-enabled brain tumor liquid biopsies [61]. Another important application will be the control of anti-tumor effect and functions of engineered immune cells such as chimeric antigen receptor T cells (CAR-T cells) within tumors. This “acoustogenetic” control, mediated by the heat generated by short pulses of FUS on a promoter for the heat-shock protein. can reversibly activate engineered T cells [62]. Besides efficacy, new future studies should evaluate carefully and exhaustively the safety profile of FUS ablation in order to compare and judge it relative to that of the more invasive but possibly proving ultimately safer Laser-Induced Interstitial Thermotherapy ablation [63,64].

## 5. Conclusions

FUS has been rarely applied for direct tumoral ablation in human HGG patients, but in current studies, it has shown some technical pitfalls and complications probably due to pioneer studies and lack of well-established inclusion criteria. Ongoing and future studies should provide data to improve patient selection and improve the risk-benefit ratio.

On the counterpart, FUS-mediated BBB opening is showing good results with very low complication rates. It seems to be a reliable technique to improve in local chemotherapy delivery and antitumoral immune response. Furthermore, this application could be complementary to surgical resection and standard radiochemotherapy in recurrent HGG. Ongoing trials should, in the near future, provide more data on FUS-mediated BBB opening impact on PFS and OS.

## Figures and Tables

**Table 1:** Demographic and pre-FUS session summary data of all-included studies.

Patient (N)	68
Age (years)	50.5 ± 15.3
Sex (M/F)	46/22
Pre-FUS surgery (N)	11
Pre-FUS radiotherapy (N)	52

**Table 2:** Demographic and pre- and post-FUS session summary data of FUS-tumor ablation studies.

Patient (N)	23
Age (years)	45.6 ± 19.4
Sex (M/F)	17/6
Pre-FUS KPS (N) *	>40
Pre-FUS surgery (N)	11
Pre-FUS RT (N)	18
Mean Sonications ^1^ (N)	30.3 ± 6.8
Post-FUS complication (%)	26.1
Follow-up ^2^ (months)	19.7 ± 14.6

* Specified only by Guthkelch et al. [44] ^1^ Guthkelch et al. [44] and Park et al. [47] did not specify. ^2^ Park et al. [47] and McDannold et al. [33] did not specify.

**Table 3:** Overall Tumor Ablation via FUS patient demographics, preoperative status and post-operative follow-up.

References	Patient (N)	Pre-FUS Treatment (Y/N, N)	Pre-FUS Debulking (N)	Pre-FUS RT (N)	Localisation	Diagnosis	Age (yy)	Sex (M/F)	Pre-FUS KPS (N)	Recurrence (Y/N, N)	HIFU Machine	Sonications (N)	Post-FUS Follow-up (Months)	Major Complications (N)	Minor Adverse Events (N)	Stopped Procedures (N)	Post-FUS KPS (N)
Guthkelch et al. 1991 [44]	15	10/5	6	10	Supratentorial	11 GBM 4 AA	61	11/4	>40	5/10	Octoson, (Ausonics Inc., Sydney, Australia)	NA	29.6	4 H (1 subcortical and 3 intracerebral) 1 TND	0	4	NA
Ram et al. 2006 [32]	3	3/0	3	3	Supratentorial	3 GBM	52	2/1	NA	3/0	ExAblate 2000 System (InSightec, Haifa, Israel)	38	26.6	1 NL	0	0	60–70
Park et al. 2006 [47]	1	1	1	1	Supratentorial	1 AA	17	0/1	NA	0/1	Mode-JC HIFU system, (Chongqing HAIFU Medical Technology Co., LTD, Chongqing, China)	NA	NA	NA	NA	0	NA
McDannold et al. 2010 [33]	3	3/0	0	3	NA	3 GBM	35	3/0	NA	3/0	ExAblate 3000 TcMRgFUS system (InSightec Ltd., Haifa, Israel)	28	NA	1 H	0	0	NA
Coluccia et al. 2014 [31]	1	1	1	1	Supratentorial	1 GBM	63	1/0	NA	1/0	ExAblate Neuro^®^ system (InSightec Ltd., Haifa, Israel)	25	3	0	0	0	NA

**Table 4:** Demographic and pre- and post-FUS session summary data of FUS-BBB Opening studies.

Patient (N)	45
Age (years)	56.6 ± 5.3
Sex (M/F)	29/16
Pre-FUS KPS (N) *	88
Pre-FUS surgery ^1^ (N)	NA
Pre-FUS RT ^2^ (N)	17
Mean Sonications ^3^ (N)	53 ± 17
Post-FUS complication (%)	0
Follow-up (months)	13.7 ± 16.1

* Specified only by Mainprize et al. [49], Idbaih et al. [51] and Chen et al. [52] ^1^ Specified only by Mainprize et al. [49] ^2^ Specified only by Carpentier et al. [48] and Idbaih et al. [51] ^3^ Specified only by Carpentier et al. [48] and Idbaih et al. [51].

**Table 5:** Overall BBB Opening via FUS patient demographics, preoperative status and post-operative follow-up.

References	Patient (N)	Pre-FUS Treatment (Y/N, N)	Pre-FUS Debulking (N)	Pre-FUS RT (N)	Localisation	Diagnosis	Age (yy)	Sex (M/F)	Pre-FUS KPS (N)	Recurrence (Y/N, N)	HIFU Machine	Sonication (N)	Post-FUS Follow-up (months)	Major Complication (N)	Minor Adverse Events (N)	Stopped Procedure (N)	Post-FUS KPS (N)
Carpentier et al. 2016 [48]	15	15/0	NA	15	NA	15 de novo GBM	62	9/6	NA	11/4	SonoCloud-9 (CarThera, Paris, France)	41	2.8	0	3 (1 Local pain 1 vagal episode 1 cerebral edema)	0	NA
Mainprize et al. 2019 [49]	5	5/0	0	No	Supratentorial	3 GBM 2 AA	56	4/1	70–100	NA	ExAblate Neuro system (InSightec Tirat Carmel, Israel)	NA	3	0	3 (1 back pain 2 Minor headache)	0	NA
Idbaih et al. 2019 [51]	19	19/0	NA	19	NA	19 GBM	59	13/6	90	19 GBM	SonoCloud-9 (CarThera, Paris, France)	65	12	0	8 (2 cerebral edema 2 vagal episode 3 facial palsy 1 sensorimotor deficit)	0	NA
Chen et al. 2021 [52]	6	6/0	NA	NA	Supratentorial	6 GBM	49.5	3/3	90	6	NaviFUS (NaviFUS Inc., Taiwan)	NA	37	0	0	0	NA

**Table 6:** FUS clinical ongoing trials characteristics and promoters.

Study	ClinicalTrials.gov Identifier	Aim	Study Location(s)	Intervention	Condition	Status
Non-Invasive Focused Ultrasound (FUS) With Oral Panobinostat in Children With Progressive Diffuse Midline Glioma (DMG)	NCT04804709	BBB Opening	Columbia University Irving Medical Center New York, NY, USA	Panobinostat 15 MG Focused Ultrasound with neuro-navigator-controlled sonication	Diffuse Intrinsic Pontine Glioma Diffuse Pontine and Thalamic Gliomas Diffuse Midline Glioma, H3 K27M-Mutant	Recruiting
Study of Sonodynamic Therapy in Participants With Recurrent High-Grade Glioma	NCT04559685	Tumor Ablation	St. Joseph’s Hospital and Medical Center, Phoenix, AZ, USA	Combination Product: SONALA-001(ALA) and MR-Guided Focused Ultrasound device (MRgFUS)	High Grade Glioma	Recruiting
ExAblate (Magnetic Resonance-guided Focused Ultrasound Surgery) Treatment of Brain Tumors	NCT01473485	Tumor Ablation	Sunnybrook Health Sciences Centre Toronto, ON, Canada	ExAblate Transcranial System	Glioma Metastatic brain cancer	Active, not recruiting
Assessment of Safety and Feasibility of ExAblate Blood-Brain Barrier (BBB) Disruption	NCT03551249	BBB Opening	University of Maryland Baltimore, MD, USA Brigham and Women’s Hospital Boston, MA, USA University of Virginia Charlottesville, VA, USA West Virginia University Morgantown, WV, USA	Focused ultrasound	Glioma Glioblastoma	Recruiting
Assessment of Safety and Feasibility of ExAblate Blood-Brain Barrier (BBB) Disruption for Treatment of Glioma	NCT03616860	BBB Opening	Sunnybrook Health Sciences Centre Toronto, ON, Canada	Focused Ultrasound BBB Disruption	Glioblastoma	Recruiting
Efficacy and Safety of NaviFUS System add-on Bevacizumab (BEV) in Recurrent GBM Patients	NCT04446416	BBB Opening	Linkou Chang Gung Memorial Hospital Taoyuan City, Taiwan	Bevacizumab NaviFUS System	Glioblastoma Multiforme Glioblastoma Glioma Brain Tumor Neoplasms Neoplasms Nerve Tissue	Recruiting
Sonodynamic Therapy With ExAblate System in Glioblastoma Patients	NCT04845919	BBB Opening and Immunologic Effect	Fondazione I.R.C.C.S. Istituto Neurologico Carlo Besta, Milan, Italy	5-Aminolevulinic Acid	Glioblastoma Multiforme	Not yet recruiting
Exablate Blood-Brain Barrier Disruption With Carboplatin for the Treatment of rGBM	NCT04440358	BBB Opening	Sunnybrook Health Sciences Centre Toronto, ON, Canada Yonsei University Medical Center Seoul, Korea	Carboplatin Exablate BBBD	Recurrent Glioblastoma	Recruiting
Exablate Blood-Brain Barrier Disruption for the Treatment of rGBM in Subjects Undergoing Carboplatin Monotherapy	NCT04417088	BBB Opening	Stanford University Palo Alto, CA, USA University of Maryland Baltimore, MD, USA Brigham and Women’s Hospital Boston, MA, USA Cleveland Clinic Cleveland, OH, USA	Carboplatin Exablate BBBD	Recurrent Glioblastoma	Recruiting
ExAblate Blood-Brain Barrier Disruption for Glioblastoma in Patients Undergoing Standard Chemotherapy	NCT03712293	BBB Opening	Severance Hospital, Yonsei University Health System Seoul, Seodaemun-gu, Korea	BBB Disruption with Chemotherapy Arm	Glioblastoma Multiforme	Recruiting
Ultrasound-based Blood-brain Barrier Opening and Albumin-bound Paclitaxel for Recurrent Glioblastoma (SC9-ABX)	NCT04528680	BBB Opening	Northwestern Memorial Hospital Chicago, IL, USA	Paclitaxel administration	Recurrent Glioblastoma	Recruiting
Innovative SonoCloud-9 Device for Blood Brain Barrier Opening in First Line Temozolomide Glioblastoma Patients. (SonoFIRST)	NCT04614493	BBB Opening	Assistance Publique—Hôpitaux de Paris—Sorbonne, Pitié Salpêtrière Hospital Paris, France Centre hospitalier Universitaire d’Angers Angers, France Groupe Hospitalier Saint-André Bordeaux, France Hospices Civils de Lyon, Hôpital Pierre Wertheimer Lyon, France AP-HM, La Timone, Hôpital Universitaire Marseille, France Centre hospitalier universitaire vaudois Lausanne, Switzerland Katholieke Universiteit Leuven Leuven, Belgium	Temozolomide administration and increasing drug delivery	Newly diagnosed Glioblastoma	Not yet recruiting
Safety and Efficacy of Transient Opening of the Blood-brain Barrier (BBB) With the SonoCloud-9 (SC9-GBM-01)	NCT03744026	BBB Opening	Assistance Publique—Hôpitaux de Paris—Sorbonne, Pitié Salpêtrière Hospital Paris, France Centre hospitalier Universitaire d’Angers Angers, France Hospices Civils de Lyon, Hôpital Pierre Wertheimer Lyon, France AP-HM, La Timone, Hôpital Universitaire Marseille, France Northwestern University Chicago, IL, USA MD Anderson Cancer Center Houston, TX, USA	Dose limiting toxicity (DLT) of number of activated ultrasound beams for carboplatin chemotherapy	Recurrent Glioblastoma	Active, not recruiting

## Data Availability

All data are available in the text.

## Abbreviations

HGG	High-grade glioma
FUS	Focused Ultrasound
BBB	Blood-Brain Barrier
US	Ultrasound
5-ALA	5-Aminolevulinic Acid
MRI	Magnetic Resonance Imaging
TMZ	Temozolomide
KPS	Karnofsky Performance Status
CT	Computed Tomography
RT	Radiotherapy
H	Hematoma
TND	Transient Neurological Deficit
NL	New Lesion
NA	Not Assessed
IV	Intravenous
GBM	Glioblastoma
AA	Anaplastic Astrocytoma
PFS	Progression-Free Survival
OS	Overall Survival
DLT	Dose Limiting Toxicity
RT	Radiotherapy
